# Ischemia/Reperfusion Injury of Fatty Liver Is Protected by A2AR and Exacerbated by A1R Stimulation through Opposite Effects on ASK1 Activation

**DOI:** 10.3390/cells10113171

**Published:** 2021-11-15

**Authors:** Elisa Alchera, Bangalore R. Chandrashekar, Nausicaa Clemente, Ester Borroni, Renzo Boldorini, Rita Carini

**Affiliations:** Department of Health Science, University of Piemonte Orientale, 28100 Novara, Italy; elisa.alchera@gmail.com (E.A.); chandrashekarbr8@gmail.com (B.R.C.); nausica.clemente@med.uniupo.it (N.C.); ester.borroni@med.uniupo.it (E.B.); renzo.boldorini@med.uniupo.it (R.B.)

**Keywords:** steatosis, ischemia/reperfusion injury, hepatic damage, oxidative stress, adenosine receptor, hepatocyte death, hepatoprotection, survival pathways

## Abstract

Hepatic ischemia/reperfusion injury (IRI) is aggravated by steatosis and is a main risk factor in fatty liver transplantation. Adenosine receptors (ARs) are emerging as therapeutic targets in liver diseases. By using cellular and in vivo systems of hepatic steatosis and IRI, here we evaluated the effects of pharmacological A2AR and A1R activation. The A2AR agonist CGS21680 protected the primary steatotic murine hepatocyte from IR damage and the activation of ASK1 and JNK. Such an effect was attributed to a phosphatidylinositol-3-kinase (PI3K)/Akt-dependent inhibition of ASK1. By contrast, the A1R agonist CCPA enhanced IR damage, intracellular steatosis and oxidative species (OS) production, thereby further increasing the lipid/OS-dependent ASK1-JNK stimulation. The CGS2680 and CCPA effects were nullified by a genetic ASK1 downregulation in steatotic hepatoma C1C7 cells. In steatotic mice livers, CGS21680 protected against hepatic IRI and ASK1/JNK activation whereas CCPA aggravated hepatic steatosis and IRI, and enhanced ASK1 and JNK stimulation. These results evidence a novel mechanism of CGS21680-mediated hepatoprotection, i.e., the PI3K/AKT-dependent inhibition of ASK1, and they show that CGS21680 and CCPA reduces and enhances the IRI of fatty liver, respectively, by preventing or increasing the activation of the cytotoxic ASK1/JNK axis. They also indicate the selective employment of A2AR agonists as an effective therapeutic strategy to prevent IRI in human fatty liver surgery.

## 1. Introduction

Ischemia/reperfusion injury (IRI) occurs as consequence of a temporary interruption of blood supply to an organ [1] and is a frequent complication of major liver surgery [2]. IRI can compromise liver function, increase postoperative morbidity and affect the overall outcome of patients [1,2]. The presence of steatosis greatly exacerbates hepatic IRI and represents a main risk factor of liver transplantation [3]. Steatosis is, in fact, a strong predictor of liver failure after transplantation, but the shortage of donors often forces the acceptance of “marginal grafts” like fatty livers [2,3]. Although hepatic steatosis is associated with an increased post-surgical mortality, no accepted therapeutic intervention is in use for the prevention of its deleterious effects [3]. 

Several pathogenic mechanisms might contribute to the increased hepatic IRI that is induced by fatty infiltration [4]. Among them, the upregulation of the mitochondrial uncoupling protein-2 with the increased production of oxidative species (OS) [5], the induction of endoplasmic reticulum stress (ERS) and the activation of inflammatory reactions play critical roles [6]. 

The Apoptosis Signal-Regulating Kinase 1 (ASK1) is a master and upstream inductor of IRI in liver and in extrahepatic tissues [7,8,9] and both OS and ER stress are among the factors that are able to induce ASK1 stimulation [7,9]. Consistently, we previously showed that the increased damage and inflammation of fatty liver that is exposed to IR is directly related to an enhanced ASK1 activation [10]. We found that the stimulation of ASK1, that is induced at the time of reoxygenation by ER stress, is increased by hepatocellular steatosis through an ROS-dependent mechanism, and we showed that such increased ASK1 activation promoted liver inflammation and exacerbated JNK-dependent hepatocyte damage [10]. These findings indicate that pharmacologic interventions targeting ASK1 stimulation are a promising approach to protect against surgically induced IRI in humans, in both normal [8,9] and fatty livers.

Adenosine is a ubiquitous nucleotide metabolite that rapidly increases within the extracellular space in several stress and distress conditions, modulating tissue function and damage [11,12]. The liver is centrally involved in nucleotide production and metabolism, and increasing evidence shows that nucleotide metabolites have a main role in modulating liver health and disease [13,14]. Adenosine, in fact, both regulates physiological liver processes like carbohydrate and lipid metabolism and acts as a damaging or protective agent, promoting or preventing different hepatic pathologies such as fibrosis, steatohepatitis, carcinogenesis and ischemia-reperfusion injury [14]. These observations focus on adenosine signaling as an attractive therapeutic target to prevent or antagonize different pathological processes in liver and in extrahepatic tissues [14,15]. 

Adenosine is signaled by activating the four G-coupled adenosine receptors (ARs): A1AR, A2AAR, A2BAR and A3A [11,12,15]. All ARs are expressed in the liver [13,14] and among them, A1AR and A2AAR display the highest affinity for adenosine [11,13]. 

In vivo and in vitro observations have shown that A2AAR activation has an established protective action against hepatic IRI [16,17,18,19,20]. A2AR stimulates multiple signal pathways that have phashatydilinositol-3-kinase (PI3K) as a key mediator, and that lead to an early and delayed resistance of primary liver cells to the death that is induced by warm and cold hypoxia and by hypoxia/reoxygenation [see for review 18,19]. Proteomic analysis of liver cells that were isolated from mice previously exposed to hepatic IR show that in vivo treatment with the A2AR agonist CGS21680 either rescues or enhances the pathways that are involved in aerobic and anaerobic energy production, which are downregulated by IR, and increases the levels of antioxidant enzymes [20]. However, the capacity of A2AR activation to also protect against IRI in fatty liver has not been yet ascertained, and the effects of A2AR signaling on the pathogenic mechanisms that are involved in the increased susceptibility of fatty liver to IRI are still unknown. 

The studies on A1R activation in hepatic IRI are fewer and the effects have been incompletely clarified. A1R blockage or loss exacerbates hepatic IRI [21,22] and A1AR agonists have demonstrated to preserve mitochondrial function in mice livers that are exposed to IR [23]. The same agonists, however, do not protect the production of hepatic IRI [21,22], suggesting that only endogenous A1R activity can promote a full development of the cytoprotective mechanisms that are needed in order to efficiently prevent hepatic IRI, whereas direct exogenous A1R stimulation fails to reproduce these effects [21]. These studies, however, only evaluated A1AR activation in the absence of steatosis. The outcomes of A1AR activation on the increased sensitivity of fatty livers to IRI are thus far undetermined. 

In this study we compared the capacity of A1AR and A2AR pharmacological activation to modulate the hypoxia/reoxygenation (HR) injury of steatotic murine hepatocytes and the IR injury of fatty livers and evaluated their effects on OS and ER stress-dependent ASK1 activation and on JNK-dependent hepatotoxicity. 

## 2. Materials and Methods

### 2.1. Animals 

Balb/c male mice were purchased at Harlan srl, Milan, Italy. All animals received human care and the study protocols were approved by the Italian Ministry of Health (authorization number: 1284/2015-PR) and by Ethical Committee of the Università del Piemonte Orientale. All animal experiments were in accordance with the “International Guiding Principles for Biomedical Research Involving Animals”.

### 2.2. Liver Cells Isolation and Treatment

Hepatocytes (HP) were isolated by liver perfusion with collagenase (Sigma, Milan, Italy) digestion [10,20]. The effect of hypoxia/reoxygenation (HR) on steatotic liver cells was investigated by employing the in vitro model of IR injury, which has been previously established [10]. Briefly, primary mouse HPs were treated for 15 min at 37 °C with palmitic acid (C16:0) (PA, 50 µM) in Krebs–Henseleit buffer and then suspended in a cold hypoxic VIASPAN solution, added to PA (50 µM), fluxed with 95% N_2_/5% CO_2_ and maintained at 4 °C for 8 h in sealed flasks. HPs were then transferred to an oxygenated Krebs–Henseleit buffer (at 37 °C), and the incubation flasks were further fluxed with a 95% air/5% CO_2_ gas mixture. When indicated, the liver cells were pre-exposed either to the A2A or to the A1 receptor agonists, CGS21680 (5 µM) and CCPA (100 µM) [18,19], and/or to the inhibitors of OS stress, DPPD (5 µM) [10] and PI3K, Wortmannin (250 nM) [24] (all from Sigma) 10 min before PA treatment. 

### 2.3. Determination of Cell Viability 

Cell viability was estimated by the determination of nuclear fluorescence staining with propidium iodide using a FACScan analyzer (Becton–Dickinson, San Jose, CA, USA) and CellQuest software (Becton–Dickinson) [10,20].

### 2.4. Measurement of Oxidant Species (OS)

Intracellular OS production was measured as previously reported [10], by quantifying the DCFH-DA (2,7-dichlorofluorescin diacetate) (Sigma) fluorescence intensity with a Hitachi F-4500 fluorescence spectrophotometer.

### 2.5. Transfection and Treatment of C1C7 Cells

The C1C7 hepatocarcinoma cell line (C1C7 cells) was obtained from the European Collection of Cell Cultures. C1C7 cells cultured on DMEM medium were transfected with 2 different murine ASK1 SiRNA (Sigma); SiRNA1 sense: CAGAUAGUCCACCGGGAUAdTdT, SiRNA1 antisense: UAUCCCGGUGGACUAUCUGdTdT; SiRNA2 sense: GUACUUCCGGGAAUCCAUAdTdT and SiRNA2 antisense: UAUGGAUUCCCGGAAGUACdTdT as previously reported [10]. In preliminary experiments, the SiRNA2 sense confirmed the best reduction of ASK1 expression [10] and was thus employed in the determinations that are reported in the Results section. The control SiRNA was used as a negative control of transfection. Cells were plated in 6-well (1.5 × 10^5^) and transfected using Lipofectamine 2000 (Invitrogen) according to the manufacturer recommendations. Transfection efficacy was analyzed after 48 or 72 h using BLOCK-iT Fluorescent Oligo (Invitrogen, San Giuliano, Milan, Italy) and was more than 75% for C1C7 cells. After SiRNA transfection (48 h), C1C7 cells were treated with 100 µM PA and then either exposed to the hypoxia/reoxygenation protocol or maintained under normoxic conditions in presence of CGS21680 or CCPA. 

### 2.6. Diet, Drug Treatment and Surgical Procedures 

The mice were fed either an isocaloric control diet (CD), or a high fat diet to induce steatosis (HFD: 58% of energy derived from fat, 18% from protein, and 24% from carbohydrates; 5.6 kcal/g) (Laboratorio Dottori Piccioni, Gessate, Milan, Italy) for 9 weeks. At the end of the dietary treatment, mice were subjected to laparotomy or to a non-lethal (−70% of the total liver volume) hepatic ischemia for 45 min, followed by 120 min of reperfusion, as previously described [14]. When indicated, they were treated by intraperitoneal injection with either sterile saline, CGS21680 (0.5 mg/kg of body weight in sterile saline) or CCPA (1.5 mg/kg of body weight in sterile saline) as previously reported [see for review: [18,19] 8 h before IR application. Each of the experimental conditions included 6 animals and the experiments were repeated 3 times. 

### 2.7. Biochemical Analysis and Liver Histochemistry and Immunohistochemistry

The serum levels of alanine aminotransferase (ALT) and liver triglycerides (TGs) were measured by using commercial assay kits (Radim, Pomezia, Italy and Sigma Diagnostics, Milano, Italy). Intracellular lipid accumulation was evaluated using the Steatosis Colorimetric Assay Kit (Cayman Chemical, St. Louis, MO, USA), according to the manufacturer’s instructions. To evaluate the intracellular lipid distribution in the cells, slides were prepared using ORO staining and visualized using a light microscope.

An immunohistochemistry analysis was performed in mice hepatic sections using a semi-automated platform, DAKO Autostainer (Dako, Carpinteria, CA, USA). ASK1 activation, which was evaluated in liver sections that were stained with the ASK1 (phospho-Thr845) antibody (Biorbyt, Cambridge, UK) (dilution 1:200), was considered positive when the staining of the HPs was detectable in the cytoplasm, independently from the intensity of the staining. Negative staining was defined a sample with background or with a staining of the interstitium. 

### 2.8. Analysis of TRAF2 Level and of the Phosphorylation State of ASK1, JNK and Akt

Protein extracts from liver pieces or liver cells were electrophoresed by SDS/PAGE (10% gel) and, after blotting them onto the nitrocellulose membranes, the membranes were probed with antibodies against TRAF2, phospho-JNK (Thr183/Tyr185), JNK, phospho-Akt (Ser473), Akt (all from Cell Signaling Technology, Danvers, MA, USA), phospho-ASK1 (Thr845) (Biorbyt), inhibitory phospho-ASK1 (Ser83) (Sigma-Aldrich) and ASK1 (Santa Cruz Technology, Santa Cruz, CA, USA). The β-actin monoclonal antibody (Sigma) was used to assess equal protein loading. The antigens were detected by Western Lightning Chemiluminescence Reagent plus (ECL) (PerkinElmer, Waltham, MA, USA) and VersaDoc 3000 quantitative imaging system (BioRad Laboratories, Milan, Italy). The results were expressed as ratios. 

### 2.9. Data Analysis

The statistical analysis was performed with InStat 3 statistical software (Graph Pad Software, Inc., San Diego, CA, USA) by a 1-way analysis of variance, testing with the Bonferroni correction for multiple comparisons when more than 2 groups were analysed. The distribution of the normality of all groups was preliminarily verified with the Kolmogorov and Smirnov test. Significance was established at the 5% level.

## 3. Results

### 3.1. Effects of A2AR and A1R Agonists on Hypoxia/Reoxygenation Damage of Steatotic Hepatocytes: Differential Modulation of Lipid Content, Oxidative Species Production and of ASK1 and JNK Activation

**Figure 1:**
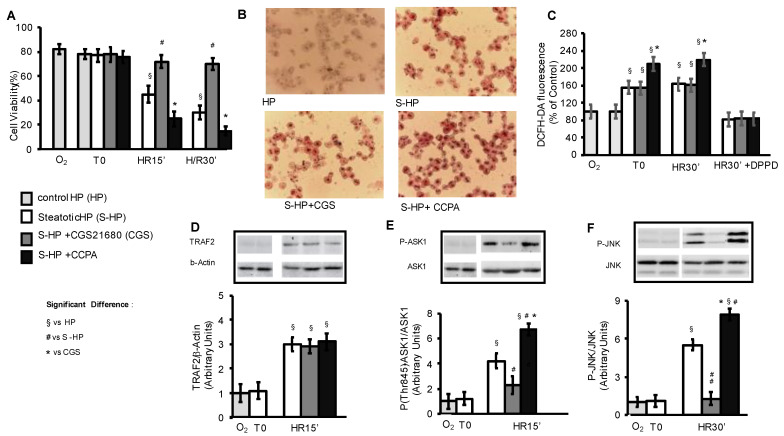
A2AR and A1AR pharmacological stimulation of primary steatotic mouse HP: effects on HR injury, OS production, TRAF2 levels and ASK1/JNK activation. Control (HP) or steatotic hepatocytes (S-HP) pre-exposed or not to the A2AR agonist CGS21680 (5 µM), to the A1R agonist CCPA (100 µM) or to the antioxidant DPPD (5 μM) were stored at 4 °C in hypoxic conditions for 8 h in presence (S-HP) or absence (HP) of palmitic acid (PA, 50 μM) and then reoxygenated at 37 °C for 30 min. (**A**) Cell viability, (**B**) Visualization of lipid intracellular content, (**C**) Oxidant species (OS) production, (**D**) TRAF2 expression, (**E**) ASK1 activation, (**F**) JNK activation. The different parameters were estimated after normoxic incubation (O2), and/or at the end of hypoxic storage (To) and after 15 and/or 30 min of reoxygenation (HR). The results are expressed as mean of 3–5 experiments ± SD. Statistical significance: * § # *p* < 0.05, ## *p* < 0.01.

We investigated the effect of the stimulation of the A1 and A2A adenosine receptors on cell damage induced by cold hypoxic storage and subsequent warm reoxygenation of steatotic primary mice hepatocytes (S-HPs) by using the pharmacological agonists of the two adenosine receptors, CCPA and CGS21680, respectively. To this end we employed the hepatocellular model of ischemia-reperfusion injury of fatty livers, which has been previously established [10]. Briefly, primary murine hepatocytes, either pre-treated with palmitic acid or not (PA, 50 µM), in either the presence or absence of CGS21680 (5 µM) or CCPA (100 µM), were preserved in hypoxic VIASPAN solution at 4 °C for 8 h and then reoxygenated in Krebs–Henseleit solution at 37 °C. CGS21680 significantly reduced the cell damage of steatotic hepatocytes that were exposed to hypoxia/reoxygenation (HR); by contrast, CCPA significantly increased it (Figure 1A). 

We previously reported that an ER stress/TRAF and an OS-dependent activation of the ASK1-JNK axis was associated with the development of the HR injury of S-HPs [10]. We also showed that the augmented susceptibility of S-HPs to HR, compared to normal primary hepatocytes, was related to a lipid-dependent increase in OS and to the consequently enhanced stimulation of ASK1 and JNK [10]. As shown in Figure 1D, neither treatment with A1 nor A2a adenosine receptor agonists, CCPA and CGS21680, modulated the TRAF2 expression that was induced by HR in S-HPs. On the other hand, CCPA significantly increased the intracellular lipid content and OS production in the S-HPs, whereas CGS 21680 did not affect them (Figure 1B,C). CCPA also augmented both ASK1 and JNK activation, whereas CGS21680 entirely prevented it (Figure 1E,F). 

These results show the capacity of the A2AR agonist CGS21680 to protect against the HR injury of steatotic hepatocytes, as well as that its protective activity is associated with a reduction in ASK1 and JNK activation. On the other hand, they reveal that the A1R agonist CCPA exacerbates the HR injury of steatotic hepatocytes and that its effect is related to an increase in the lipid hepatocellular content and in the OS production, as well as to a further stimulation of ASK1 and JNK activity.

### 3.2. Role of ASK1 in the Cytoprotective and Cytotoxic Effects of the A2AR and A1R Agonists: Effect of the Genetic Silencing of ASK1

**Figure 2:**
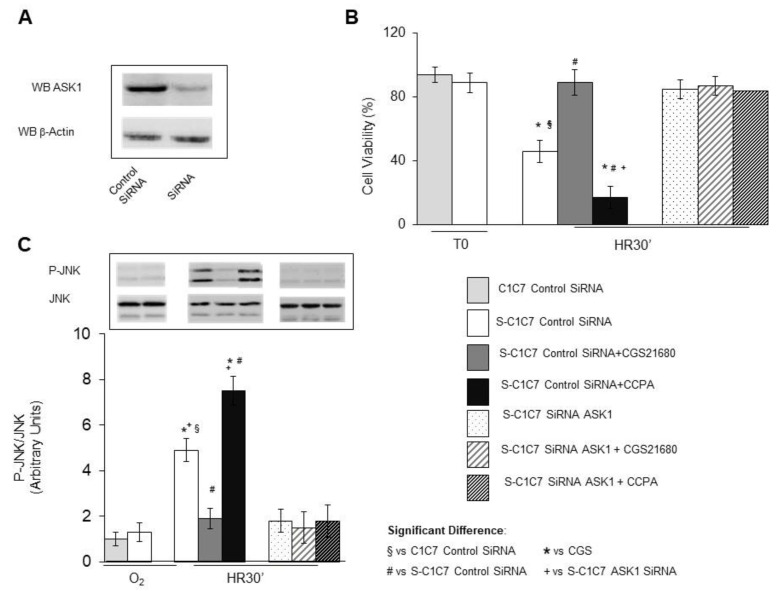
Effects of A2AR and A1AR pharmacological stimulation on HR injury and ASK1/JNK activation of steatotic C1C7 cells with downregulated expression of ASK1. Steatotic (PA 700 μM, S-C1C7) and non steatotic C1C7 cells (C1C7) transfected with control SiRNA or ASK1 SiRNA and pre-exposed or not to the A2AR agonist CGS21680 (5 µM) or to the A1R agonist CCPA (100 µM), were stored at 4 °C for 8 h in hypoxic conditions in in presence (S-C1C7) or absence (C1C7) of palmitic acid (PA, 700 μM) and reoxygenated at 37 °C for 30 min (HR30′). (**A**) WB analysis of ASK1 expression after C1C7 cells transfection with Control SiRNA or ASK1 SiRNA (SiRNA); (**B**) Cell viability; (**C**) JNK activation. The different parameters were estimated after normoxic incubation (O2), and/or at the end of hypoxic storage (T0), or after 30 min of reoxygenation (HR). The results represent the mean of 3 experiments ± SD. Statistical significance: * # § *p* < 0.05.

To investigate the role of ASK1 in mediating the outcomes of the A2AR and A1 agonists CGS21680 and CCPA on HR damage, we evaluated their effects upon the depletion of ASK1 (Figure 2). To this end we employed both mice steatotic hepatoma cells C1C7 (S-C1C7 control SiRNA) with and without silenced ASK1 expression (S-C1C7 SiRNA ASK1) [10]. As shown in Figure 2B,C, neither CGS21680 nor CCPA affected the prevention of HR damage and JNK activation that is associated with ASK1 silencing; instead, both CGS21680 and CCPA confirmed their capacity to protect against and increase, respectively, HR damage and JNK activation in S-C1C7 cells that were transfected with control siRNA, as observed in primary hepatocytes. 

These data show the critical role of ASK1 in the protective and toxic activity of the A2AR and A1R agonists, respectively.

### 3.3. Mechanisms Involved in the Cytoprotective and Cytotoxic Activity of the A2AR and A1R Agonists: Effects of the Inhibition of PI3K/Akt and of OS Production

**Figure 3:**
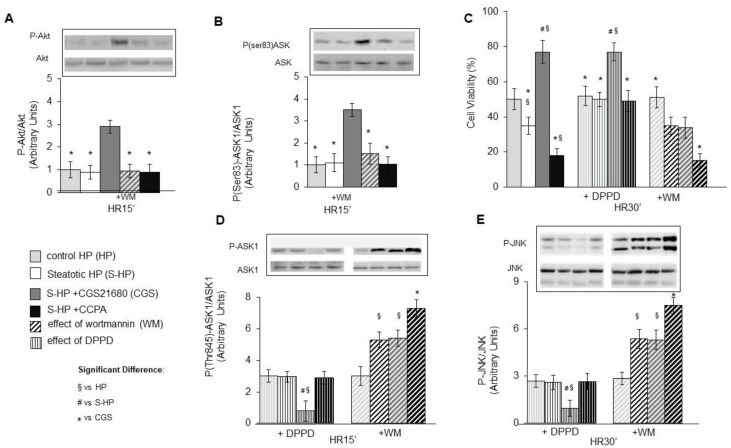
Inhibition of OS production or of PI3K/Akt activation in steatotic HP treated with A2AR or A1R agonist and exposed to H/R: effect on viability, on activation of Akt, ASK1 and JNK and on inhibition of ASK1. Control (HP) and steatotic hepatocytes (S-HP) pre-exposed or not to the A2AR agonist CGS21680 (5 µM) or to the A1R agonist CCPA (100 µM), in presence or absence of the antioxidant DPPD (5 μM) or of the PI3K/Akt inhibitor wortmannin (WM, 5 nM), were stored at 4 °C for 8 h in hypoxic VIASPAN solution added (S-HP) or not (HP) with palmitic acid (PA, 50 μM) and then reoxygenated at 37 °C for 30 min. (**A**) Akt activation. (**B**) ASK1 inhibition, (**C**) Cell viability, (**D**) ASK1 activation, (**E**) JNK activation. The different parameters were estimated after 15 or 30 min of reoxygenation (H/R). The results are expressed as mean of 3–5 experiments ± SD. Statistical significance: * # § *p* < 0.05.

Several signals can positively or negatively modulate ASK1 activity, and among them, Akt was proven to directly prevent ASK1 activation by inducing ASK1 phosphorylation on Ser 83 [24]. PI3K/Akt axis activation has a central role in mediating the cytoprotective effects of the A2a adenosine receptor stimulation in primary hepatocytes [25]. Hence, we evaluated the PI3K/Akt activation as the phosphorylation of the PI3K downstream mediator Akt in steatotic hepatocytes that were exposed to HR. We observed that A2A receptor stimulation with CGS21680 significantly increased Akt phosphorylation, whereas A1 receptor stimulation with CCPA did not affect it (Figure 3A). We then analyzed the levels of the phosphorylation of Ser 83 on ASK1 in steatotic hepatocytes that were subjected to HR, in the presence or absence of CGS21680 or CCPA. As shown in Figure 3B, HR alone or HR plus CCPA did not induce ASK1 phosphorylation on Ser 83. On the contrary, CGS21680 significantly increased the inhibitory phosphorylation of ASK1 that was prevented by the PI3K inhibition with Wortmannin (WM) (Figure 3B). PI3K inhibition also abolished the protective effects of CGS21680 on the cell damage (Figure 3C) and on ASK1 and JNK activation (Figure 3D,E). 

To investigate the role of the increased production of OS species that is induced by CCPA, we evaluated the effects of the treatment with the antioxidant DPPD (5 µM). In accordance with what was previously reported [10], DPPP entirely prevented OS production (Figure 1C) and abolished the increase of HR damage and of ASK1/JNK stimulation of S-HPs compared to HPs, and it also nullified the enhancement of the toxic effects that were induced by CCPA (Figure 3C–E). On the other hand, DPPD did not affect the protection of CGS21680 against hepatocyte damage and against ASK1 and JNK activation (Figure 3C–E).

These observations enlighten the capacity of the A2AR agonist CGS21680 to exert its protective effects against the HR damage of steatotic hepatocytes by promoting an Akt/PI3K-dependent inhibition of ASK1. On the other hand, they show that the increased damage of steatotic hepatocytes exposed to HR that is induced by the A1R agonist CCPA are mediated by an enhanced OS production with consequent and further activation of the cytotoxic ASK1/JNK pathway. 

### 3.4. In Vivo Studies

**Figure 4:**
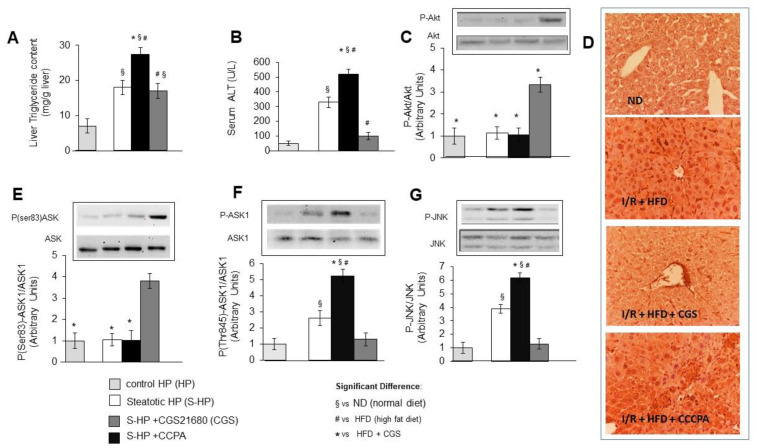
Effects of pharmacological A2AR and A1R stimulation on hepatic IRI of steatotic mice. Mice fed with normal (ND) or high fat diet (HFD) for 9 weeks, treated or not by I.P. injection with the A2aR agonist, CGS21680 (0.5 mg/kg) or with the A1R agonist CCPA (1.5 mg/kg), were subjected to 45 min of ischemia followed by 120 min of reperfusion (IR). (**A**) Hepatic triglyceride content (**B**) ALT release (**C**) Akt activation (**D**) Visualization of activated ASK1 (**E**) ASK1 inhibition; (**F**) ASK1 activation; (**G**) JNK activation. The results represent the mean of 3 experiments ± SD. Statistical significance: * # § *p* < 0.05.

To analyze the in vivo effects of the A1R and A2AR agonists, we employed mice that were fed with either a standard diet or a high fat diet (HFD) for 9 weeks, either treated or not with with CCPA (i.p. 1.5 mg/Kg) or CGS21680 (i.p. 0.5 mg/kg), and then exposed to liver ischemia/reperfusion. Hepatic steatosis was induced by feeding with a HFD, was evaluated using the liver triglyceride content, was not affected by CGS21680 treatment, but was significantly increased by CCPA (Figure 4A). In HFD-fed mice, CGS21680 treatment protected against liver damage, which was evaluated using serum ALT releases (Figure 4B), increased the phosphorylation of Akt (Figure 4C) and of Ask1 in Ser 83 (Figure 4E) and reduced both Ask1 (Figure 4D,F) and JNK activation (Figure 4G). On the contrary, CCPA treatment exacerbated ALT release (Figure 4B), did not induce Akt or Ser 83 ASK1 phosphorylation (Figure 4C,E), and augmented the activation of Ask1 (Figure 4D,F) and JNK (Figure 4G). 

These in vivo findings demonstrate the capacity of the pharmacological A2AR stimulation by the A2AR agonist CGS21680 to efficiently protect against the IRI damage of steatotic mice liver. They also confirm that A2AR hepatoprotective activation is associated with the stimulation of the survival mediator Akt and with the prevention of ASK1 and JNK activity. By contrast, these in vivo results reproduce the toxic effects of the A1R pharmacological activation by CCPA, showing its capacity to aggravate the IRI damage of steatotic mice liver and evidencing its pro-steatogenic effects and its exacerbation of ASK1 and JNK activity. 

## 4. Discussion

Hepatic steatosis is usually an asymptomatic condition, but it represents the single main risk factor in major liver surgery and transplantation [3]. Therapeutic interventions that are able to reduce and protect against the deleterious consequences of IRI in fatty livers are therefore urgently needed. 

The present study analyzed the therapeutic potential of A1R and A2AR pharmacological activation and shows that the A2AR agonist CGS21680 significantly protects against the HR damage of primary hepatocytes and the IRI of fatty livers. By contrast, the A1R agonist CCPA fails to produce protective effects and even exacerbates the HR injury of steatotic hepatocytes and the IRI of fatty livers. 

The evaluation of the mechanisms that are responsible for the divergent outcomes of the two AR agonists evidenced that CCPA promotes, both in primary hepatocytes and in the liver, an increased lipid accumulation, whereas CGS21680 does not affect it. Interestingly, previous studies with mice that were genetically deleted of A1AR or A2AR have already reported an opposite function of the endogenous activation of A1R and A2AR on lipid metabolism. Early studies from Peng et al. showed that A1ARKO mice were protected from developing ethanol-induced fatty livers by preventing the increase of the enzymes that are involved in fatty acid synthesis induced by ethanol [26]. Cai et al. instead found that an A2AR deficiency increased the severity of the HFD-induced hepatic steatosis by suppressing the A2AR-induced repression of SREBP1c (sterol regulatory element-binding protein 1c), which is a key inductor of hepatic lipogenesis [27]. These results suggest the pro-steatogenic and anti-steatogenic roles of A1R and A2AR activity, respectively, and our observations on the effects of direct exogenous A1AR and A2AR activation appear consistent with these roles.

Several studies correlate intracellular steatosis with the promotion of oxidative stress [5,6] and we previously showed that the increased ASK1 activation in the S-HPs that were exposed to HR depends on the increase in the oxidative stress that is associated with lipid accumulation [10]. We consistently demonstrated here that the exacerbation of ASK1 activation induced by CCPA was related to the augmented intracellular steatosis and to an enhanced OS production, and that OS prevention abolished the increased toxic effects of CCPA. 

ASK1 signaling is modulated by multiple factors and stressful conditions and oxidative species are among the damaging conditions that have been proven to stimulate ASK1 [7,8,9]. Several proteins are also known to interact with ASK1 and to negatively or positively regulate its activity [7,8,9]. Among them, PI3K/Akt, the central signal mediators of the survival pathway that is activated upon A2AR activation [19,24], has been established to decrease ASK1 activity by phosphorylating a consensus Akt site at serine 83 [25]. Accordingly, we confirmed the capacity of A2AR to also promote Akt activation in S-HPs that were exposed to HR and found that it was associated with the inhibitory phosphorylation of ASK1 and with the prevention of its activation. By contrast, the A1AR agonist proved to be incapable of activating Akt and did not produce the protective inhibition of theASK1 activity and instead promoted the increased ASK1 activation resulting from the enhanced lipid content and the consequently augmented OS production. 

ASK1 is a member of the mitogen-activated protein kinase kinase kinase (MAP3K) family that activates the downstream MAP kinase kinases 4 and 6 and the stress-activated kinase c-Jun N-terminal kinases (JNKs) [28], which is an effector of hepatic IRI in both lean and fatty liver [29].

Consistently, we observed that A2AR-induced hepatoprotection relied on the prevention of ASK1 and JNK activation, whereas the exacerbation of damage induced by A1AR was associated with an enhanced stimulation of the ASK1/JNK1 axis. Thus, the differential effects of the A1AR and A2AR agonists on ASK1 activation appear to be the central mechanisms responsible for their opposite modulation of the IRI of fatty livers. 

Direct ASK1 or JNK inhibition can efficiently prevent hepatic IRI in lean or fatty livers in animal models [10,29]. The employment of inhibitors of ASK1 or JNK with human patients would be, however, problematic because of the multiple physio-pathological roles of these kinases. ASK1 acts, in fact, as a broad sensor of potentially damaging conditions and its blockage would induce a general inhibition of tissue capacity to respond to different stresses [7,8,9]. On the other hand, JNK was proven to exert toxic but also pro-survival effects and its blockage would avoid the protective outcomes of its activity [9,10]. In a clinical approach it would thus be safer to target the ASK1/JNK activation in the isolated trial of a specific pathology.

The results of the present study show that the A2AR agonist CGS21680 can efficiently prevent the IRI of fatty livers by inhibiting the IR- and lipid-induced stimulation of the ASK1/JNK axis. These observations address the A2AR activation as a useful approach to attribute the blockage of ASK1 to its protective action on hepatic IRI and point to the A2AR agonists as suitable therapeutic agents for the prevention of the IRI of fatty livers. 

In recent decades, several methods, mostly targeting singular IR-induced alterations, were found to be able to reduce the IRI in both lean and fatty livers in animal models [2,4]. Among them, ischemic preconditioning (IP), which is induced by a short interruption of blood flow followed by re-perfusion [30], emerged due to its powerful and multifaced beneficial effects [18,19,31,32]. Surgical IP is a simple and inexpensive technique, and therefore it is easily applicable in ordinary hepatic surgery [32]. IP also proved to reduce the IR damage of both lean and fatty livers in both preclinical and clinical pilot studies [31,32,33,34]. The routine application of IP, however, has by now been discouraged due to the low efficacy of this procedure that was reported by singular clinical studies [34]. The inter-individual variability, as well as the different clinical settings or patient conditions, may explain these observations [34]. 

One possible approach to overcome the limits of IP efficacy would be the potentiation of its protective activity with a direct pharmacological stimulation of critical IP mediators. IP application, in fact, activates a complex and only partially elucidated network of signal pathways that can contribute differently to its protective effects. It is thus conceivable that the selective stimulation of an established cyto-protective pathway could avoid the molecular circuits that are probably affecting or decreasing IP efficacy. 

Since the earliest studies on hepatic IP [16,17,35,36], in vivo and in vitro observations have shown that IP increases the extracellular adenosine levels that, in turn, triggers the IP protective effects upon the stimulation of A2AR of liver cells. Several studies have proven that the pharmacological or genetic inhibition of A2AR prevents the beneficial effects of IP [16,17,19,35], and A2AR activation is thus generally regarded as the main inductor for IP-induced liver protection against IR injury. Adenosine that is released following IP application is, however, likely to interact with the other ARs that are expressed in the liver and, among them, A1R and A2AR are the receptors with the highest affinity for adenosine. 

This study shows that A1R activation exacerbates the HR damage of steatotic hepatocytes and the IRI of steatotic livers, thereby increasing hepatic lipid accumulation and enhancing the activation of the ASK1/JNK pathway. Although dedicated research is needed in order to investigate the outcomes of A2AR and A1R inhibition upon IP application in experimental models of fatty liver transplantations, the present study indicates that conditions leading to A1R stimulation can result in an enhancement of IRI in fatty liver and that A2AR agonists might effectively improve the protective effects of surgical IP by avoiding the detrimental consequence of the A1R activation in the specific procedure of steatotic liver surgery. Additionally, specific studies will be required in order to evaluate the impact of A1R and A2AR activation in the presence of different grades of hepatic steatosis. A low degree of steatosis, in fact, might be difficult to identify in patients and the methods that are available for the appropriate detection are invasive and thus not often routinely employed. 

## 5. Conclusions

The present study shows that pharmacological stimulation of the two adenosine receptors with the highest affinity for adenosine, A1 and A2A, display opposite effects on the IRI of murine fatty livers. A1R stimulation aggravates IRI, thereby increasing hepatic steatosis and thus further promoting the lipid/OS-dependent stimulation of ASK1 and JNK. A2AR stimulation protects against IRI by an Akt-mediated inhibition of ASK1 activation. These observations show the capacity of A2AR activation to also prevent hepatic IRI in the presence of steatosis and enlighten a novel survival circuit that is implicated in the hepatoprotective action of A2aR: the PI3K/Akt-induced prevention of the cytotoxic ASK1/JNK signaling. These observations support the potential of the therapeutic employment of A2AR agonists to antagonize the damaging effects of IR upon fatty liver surgery and suggest that selective A2AR targeting can represent a more efficient therapeutic approach compared to the conditions that are also potentially able to stimulate A1R, such as ischemic preconditioning. 

## Data Availability

Not applicable.
